# Editorial: Higher-Order Conditioning: Beyond Classical Conditioning

**DOI:** 10.3389/fnbeh.2022.928769

**Published:** 2022-05-25

**Authors:** Arnau Busquets-Garcia, Nathan M. Holmes

**Affiliations:** ^1^Cell-Type Mechanisms in Normal and Pathological Behavior Research Group, Neuroscience Programme, IMIM Hospital del Mar Medical Research Institute, Barcelona, Spain; ^2^School of Psychology, University of New South Wales, Sydney, NSW, Australia

**Keywords:** higher-order conditioning, sensory preconditioning, second-order conditioning, rodent model, humans

## Introduction

Pavlovian conditioning is the means by which animals learn about cues that signal biologically significant events, such as the presence of food or danger. In the laboratory, it is studied in a range of species, including fish, crabs, snails, birds, rodents, primates, and human subjects. Studies of so-called “higher-order” Pavlovian conditioning have provided specific insights to how animals learn about cues that signal innocuous events, how different types of associations are linked in a memory network, and how memories are retrieved from a network to guide behavior. Such studies are rapidly gaining popularity in the field of behavioral neuroscience. They have the potential to accelerate our understanding of how learning and memory is organized in the brain, and thereby, disturbances of learning and memory that underlie various brain pathologies.

This Research Topic consists in a series of empirical and theoretical papers that analyze the two types of higher-order conditioning: sensory preconditioning and second-order conditioning. These papers specifically address what is learned in sensory preconditioning and second-order conditioning, and how this learning is expressed in behavior; the pharmacological and neural processes that regulate the two types of conditioning; and points of contact between studies of higher-order conditioning in animal and human subjects.

The first set of papers addresses what is learned during sensory preconditioning and second-order conditioning; and how this learning is retrieved/expressed in behavior. Prével and Krebs review findings that the level of responding to a sensory preconditioned or second-order conditioned stimulus can be independent of the level of conditioning to its first-order conditioned stimulus-associate; and consider implications of this independence for classic and contemporary theories of learning and memory. Gostolupce et al. review factors that influence sensory preconditioning and second-order conditioning; what is learned in different types of sensory preconditioning and second-order conditioning protocols, and how an appreciation of these differences might help to identify the functions of specific brain regions. Honey and Dwyer provide a formal analysis of higher-order conditioning according to their model, HeiDI (How excitation and inhibition determine ideo-motion), which attributes sensory preconditioned and second-order conditioned responding to complex (but principled) chains of associations that form in training; that is, they explicitly address how the two forms of higher-order conditioning are expressed in behavior. Finally, Muñiz-Diez et al. show that, when second-order conditioning is established using a feature negative discrimination across multiple training sessions, the second-order stimulus initially elicits responding, gradually ceases to elicit responding and eventually passes a retardation test for the presence of inhibition.

The second set of papers examines/reviews the pharmacological underpinnings of sensory preconditioning and second-order conditioning. It shows that associative formation between affectively neutral stimuli in sensory preconditioning is enhanced by a systemic injection of an opioid receptor antagonist (Michalscheck et al.); impaired by a systemic injection of a dopamine receptor antagonist (D1 or D2; Roughley et al.); and also impaired by a systemic or intra-hippocampal injection of a cannabinoid receptor antagonist (CB1; Ioannidou et al.). It also shows that midbrain dopamine regulates appetitive second-order conditioning in the same way that it regulates associative formation in sensory preconditioning (Seitz et al.); and opioid receptor-dependent signaling regulates aversive second-order conditioning in the same way that it regulates associative formation in sensory preconditioning (Michalscheck et al.). Taken together, these studies imply that: (1) despite their co-evolution, endocannabinoid and opioid receptors influence sensory preconditioning in different ways; (2) despite their differences, sensory preconditioning and second-order conditioning share the same pharmacological underpinnings in the brain; (3) given the links between error-correction and midbrain dopamine in appetitive protocols, error-correction drives the learning that occurs in appetitive second-order conditioning; and (4) given the links between error-correction and opioid receptor-signaling in aversive protocols, error correction drives the learning that occurs in aversive second-order conditioning.

Fournier et al. extend this analysis by reviewing the brain regions that are engaged during sensory preconditioning and/or second-order conditioning. These regions include the hippocampus (Busquets-Garcia et al., 2018), amygdala (Parkes and Westbrook, 2011), orbitofrontal, perirhinal, and retrosplenial cortices (Robinson et al., 2014; Holmes et al., 2018; Sadacca et al., 2018). Fournier et al. focus on the retrosplenial cortex which is shown to encode sensory preconditioned associations in the absence of reinforcement. On the basis of these demonstrations, they argue that future work should examine how the retrosplenial cortex interacts with other regions during sensory preconditioning as this will shed light on how this brain region encodes and stores very basic types of information. More generally, it will be important to assess how the aforementioned brain regions interact with each other during both forms of higher-order conditioning as this will lay a foundation for discovering how the brain encodes and stores different types of information.

The remaining papers in our Research Topic include studies of higher-order conditioning in people. While higher-order conditioning has been demonstrated many times in animal subjects (Gewirtz and Davis, 2000), there are relatively few demonstrations in humans; so much so that it has been described as experimentally elusive in these subjects. In this respect, Lee recognizes difficulties in performing second-order conditioning experiments in humans and identifies critical parameters for establishing reliable effects in these subjects. Dhamija et al. provide a novel demonstration of second-order conditioning in humans using electrophysiological responses as a measure of performance; and some evidence that first- and higher-order conditioning might be supported by different neural substrates. Bouchekioua et al. review evidence that has been taken to indicate the use of reasoning-like processes when navigating in a new environment; and show that goal-directed navigation can be explained as the result of higher-order associative learning rather than by appeal to reason or inference. Finally, Wang et al. examine the episodic-like basis of sensory preconditioning in human subjects; and suggest that distinct memories might be manipulated to achieve better outcomes in the treatment of various pathologies [e.g., post-traumatic stress disorders (PTSD)].

## Concluding Remarks

Second-order conditioning and sensory preconditioning were first-described many decades ago: the former by Pavlov (1927) and the latter by Brogden (1939). It is now recognized that the study of higher-order conditioning has the potential to answer fundamental questions about how the brain processes and integrates different types of information; and that the answers to these questions will advance our understanding of how the brain works under normal and pathological conditions. However, two major gaps must be addressed before such understanding can be attained. First, it will be important for higher-order conditioning to be reliably established in laboratory studies with human subjects, as this will expand analysis of the ways in which environmental stimuli influence decision making and contribute to psychopathology. Second, we must improve the dialogue between researchers that study higher-order conditioning in animals and clinicians that treat psychopathology (including PTSD, addictions and anxiety disorders) to ensure that gains in knowledge from basic science research are useful and applied in the development of therapeutic strategies.

All of this is to say that, while our understanding of the behavioral, pharmacological and neural substrates of higher-order conditioning has advanced over the past few decades, much work remains to be done. Our Research Topic identifies lines of inquiry that could and should be pursued; and, ultimately, how theories of higher-order conditioning might be grounded in the brain and used to inform the management/treatment of psychopathology.

## Author Contributions

AB-G and NH have contributed equally to this work and approved it for publication.

## Funding

This work was supported by the IBRO Return Home Fellowships 2019 and the ERC Starting Grant (HighMemory, #948217) received by AB-G. AB-G is a Ramon y Cajal researcher (RYC-2017-21776, funded by MCIN/ AEI/10.13039/501100011033 and FSE). Finally, NH was supported by an Australian Research Council (ARC) Future Fellowship (FT190100697).

## Conflict of Interest

The authors declare that the research was conducted in the absence of any commercial or financial relationships that could be construed as a potential conflict of interest.

## Publisher's Note

All claims expressed in this article are solely those of the authors and do not necessarily represent those of their affiliated organizations, or those of the publisher, the editors and the reviewers. Any product that may be evaluated in this article, or claim that may be made by its manufacturer, is not guaranteed or endorsed by the publisher.

## References

[B1] Brogden W. J. (1939). Sensory pre-conditioning. J. Exp. Psychol. 25, 323–332. 10.1037/h0058465 18920626

[B2] Busquets-Garcia A. da Cruz J. F. O. Terral G. Pagano Zottola A. C. Soria-Gómez E. Contini A.. (2018). Hippocampal CB1 receptors control incidental associations. Neuron 99, 1247–1259 e1247. 10.1016/j.neuron.2018.08.014 30174119

[B3] Gewirtz J. C. Davis M. (2000). Using pavlovian higher-order conditioning paradigms to investigate the neural substrates of emotional learning and memory. Learn Mem. 7, 257–266. 10.1101/lm.35200 11040256

[B4] Holmes N. M. Raipuria M. Qureshi O. A. Killcross S. Westbrook F. (2018). Danger changes the way the mammalian brain stores information about innocuous events: a study of sensory preconditioning in rats. eNeuro 5:2017. 10.1523/ENEURO.0381-17.2017 29464195 PMC5815846

[B5] Parkes S. L. Westbrook R. F. (2011). Role of the basolateral amygdala and NMDA receptors in higher-order conditioned fear. Rev. Neurosci. 22, 317–333. 10.1515/RNS.2011.025 21591909

[B6] Pavlov I. P. (1927). Conditioned reflexes: an investigation of the physiological activity of the cerebral cortex. Ann. Neurosci. 17, 136–141. 10.5214/ans.0972-7531.1017309 25205891 PMC4116985

[B7] Robinson S. Todd T. P. Pasternek A. R. Luikart B. W. Skelton P. D. Urban D. J.. (2014). Chemogenetic silencing of neurons in retrosplenial cortex disrupts sensory preconditioning. J. Neurosci. 34, 10982–10988. 10.1523/JNEUROSCI.1349-14.2014 25122898 PMC4131013

[B8] Sadacca B. F. Wied H. M. Lopatina N. Saini G. K. Nemirovsky D. Schoenbaum G. (2018). Orbitofrontal neurons signal sensory associations underlying model-based inference in a sensory preconditioning task. eLife 7:e30373. 10.7554/eLife.30373 29513220 PMC5847331

